# Casing Contribution to Proteolytic Changes and Biogenic Amines Content in the Production of an Artisanal Naturally Fermented Dry Sausage

**DOI:** 10.3390/foods9091286

**Published:** 2020-09-13

**Authors:** Annalisa Serio, Jessica Laika, Francesca Maggio, Giampiero Sacchetti, Flavio D’Alessandro, Chiara Rossi, Maria Martuscelli, Clemencia Chaves-López, Antonello Paparella

**Affiliations:** Faculty of Bioscience and Technology for Food, Agriculture and Environment, University of Teramo, via Balzarini 1, 64100 Teramo, Italy; aserio@unite.it (A.S.); jessica.laika@hotmail.com (J.L.); fmaggio@unite.it (F.M.); gsacchetti@unite.it (G.S.); flavio_dale@live.it (F.D.); crossi@unite.it (C.R.); mmartuscelli@unite.it (M.M.); apaparella@unite.it (A.P.)

**Keywords:** proteolysis, dry fermented sausage, casing, biogenic amines, volatile compounds, texture, low temperature

## Abstract

The effect of two kinds of casings on the production and characteristics of a dry fermented sausage was investigated. In detail, an Italian product, naturally fermented at low temperatures and normally wrapped in beef casing instead of the most diffused hog one, was selected. Two different productions (one traditionally in beef casing (MCB) and another in hog casing (MCH)) were investigated over time to determine the differences particularly regarding proteolytic changes during fermentation and ripening. First of all, the product in hog casing required a longer ripening time, up to 120 days, instead of 45–50 days, because of the lower drying rate, while the microbial dynamics were not significantly modified. Conversely, the proteolysis showed a different evolution, being more pronounced, together with the biogenic amines content up to 341 mg/Kg instead of 265 mg/Kg for the traditional products. The latter products were instead characterized by higher quantities of total free amino acids, 3-methyl butanoic acid, 3-Methyl-1-butanal, and 2-Methylpropanal, enriching the final taste and aroma. The traditional product MCB also showed lower hardness and chewiness than MCH. The results highlight how the choice of casing has a relevant impact on the development of the final characteristics of fermented sausages.

## 1. Introduction

The characteristics of dry fermented sausages depend on many factors, including the ingredients, the recipe, the microbiota composition, and the different production steps. These factors, combined together, determine the variety of products widespread in different European countries [1]. Many fermented meats are produced in Italy; among them, a very particular one is produced during autumn and winter in the Abruzzo Region (Central Italy), and is called “*Mortadella di Campotosto*”. It is made of lean pork meat mixed with salt, pepper, and nitrite and it contains a bar of lard, previously cured with salt and spices for about two weeks. It shows a characteristic sub-ovoid shape and length about 15–20 cm, with a diameter of 8–10 cm. Among the typical characteristics, this product is handmade and wrapped (instead of being stuffed) into a natural beef casing; afterwards, it is fermented at low temperature without any starter addition. Traditionally, the product is exposed to dry cold northern winds that provide the ideal conditions for product ripening, which lasts about 40–50 days, with temperatures varying from −1 °C to about 10 °C.

As for this product, the traditional casing is made of beef middles, we decided to evaluate the effect of both the traditional casing and a more usual hog casing on the characteristics of the sausage. In fact, although “*Mortadella di Campotosto*” sausage manufacturing does not have any particular specifications about the natural casing to use, most of the producers use beef casing, but some may use hog casing because of its larger availability.

As regards fermented sausages, besides containing the meat batter, the casing exerts mechanical protection and guarantees permeability, which is the basis of the exchange of water and oxygen necessary for adequate and homogeneous drying and ripening [2]. Many features affect the quality of natural casings, such as the portion of the intestinal tract used, the manipulation of the product [3] and the mechanical and physical characteristics such as casing elasticity, permeability to water and gases, diameter and its uniformity, adhesion, and resistance to temperature variations [2]. In addition, casing can be a relevant source of enzymes, while casing microbiota is important overall when starter cultures are not added for fermentation [4].

In light of these considerations, we hypothesized that different casings could affect ripening by modifying sausage microbiota, water diffusion, and proteolysis dynamics, with a potentially significant impact on the product quality. Therefore, in this research, we aimed to deepen the knowledge on the influence of the type of casing on the characteristics of “*Mortadella di Campotosto*”-like sausages and to highlight the effect of casing on specific quality traits of the product. For these reasons, the specific objectives were: to follow the evolution of the principal microbial groups during the production process; to evaluate the protein hydrolysis of the fermented meats soon after stuffing and during ripening; to evaluate the texture and flavor of the finished product; and to follow the production of biogenic amines during the process.

## 2. Materials and Methods

### 2.1. Samples Production

“*Mortadella di Campotosto*”-like sausages were manufactured in autumn–winter in Macelleria “L’Olmo”, sited in Scanno (AQ), Italy. The sausages were produced according to the traditional formulation and procedure: pork lean meat (ham, shoulder, and loin) and bacon were first minced, then mixed with salt (20 g/Kg), black pepper (1.0 g/Kg), and a mixture of sucrose, glucose, ascorbic acid (E300), and potassium nitrite (E252) in a total quantity of 4.0 g/Kg. After 12 h at 4 °C, the meat mix was then minced again, and about 420 g was taken for each sausage. A stripe of back lard was previously cured at 4 °C with salt, spices (pepper, oregano, rosemary, pimento, juniper, and cilantro), and a mixture of sucrose, glucose, ascorbic acid, and potassium nitrite, for about 14 days, after which it was cut into portions of about 3 × 3 × 10 cm that were inserted into the batter. Then, the product was shaped in its typical oval form. Successively, the meat balls were hand-wrapped with the natural casing, previously washed with water and vinegar, then air-dried for about 10 h. Then, the products were left in air for 3–4 h, to favor the sealing of the casing edges; afterward, they were linked with a cotton string of medium caliber and tied in pairs. Thirty-one samples for each batch were used. The size of the samples at T0 was 17 ± 2 cm in length × 9 ± 2 cm in width and the weight was 452 ± 18 g. At the end of ripening, the size was about 10 ± 1 cm in length × 6 ± 1 cm in width and about 20 ± 1.5 in diameter and a final weight of about 270 ± 10 g.

Two different batches were produced: batch A, named MCB, wrapped with beef casing (beef middles)**,** and batch B, named MCH, wrapped with hog casing (hog bung). All samples were transferred to a fermentation room, in which the temperature varied from −2 and 5 °C for 5 days. The samples were then moved to a drying–ripening room, where they stayed up to 120 days. In the room, natural ventilation was favored, with variable relative humidity (depending on the external weather conditions) and temperatures below 12 °C.

### 2.2. Microbiological Analysis

Microbiological analyses were carried out on the batter section, excluding the lard, after casing removal. Ten grams of the sausages were homogenized with 90 mL of sterile 0.1% (*w/v*) peptone water for 2 min, using sterile plastic pouches in a Stomacher Lab-Blender 400 (PBI International Milan, Italy). Serial 10-fold dilutions were prepared in sterile peptone water solution and inoculated in duplicate in appropriate culture media. The following microbial groups were determined: aerobic mesophilic bacteria on Plate Count Agar (PCA) at 30 °C for 48 h; mesophilic lactobacilli (LAB) and cocci on MRS agar and M17, respectively, at 30 °C for 48–72 h under anaerobiosis; presumptive enterococci on Slanetz and Bartley agar (S&B) at 37 °C for 48 h; total enterobacteria on Violet Red Bile Glucose Agar (VRBGA) at 37 °C for 24 h; micrococci and staphylococci on Mannitol Salt Agar (MSA) at 30 °C for 72 h; yeasts on Yeast extract-Peptone-Dextrose agar (YPD), added with 150 ppm of chloramphenicol, at 30 °C for 72 h.

Mold development on the casing was evaluated by sampling 10 cm^2^ of casing and determining mold growth on YPD added with 150 ppm of chloramphenicol. All the culture media were from Oxoid SpA (Rodano, Italy).

### 2.3. Physical Analyses

Measurement of water activity (a_w_) was performed by the Aqualab instrument CX/2 (Series 3, Decagon Devices, Inc., Pullman, WA, USA). Samples (10 g) were randomly obtained from the sausage (batter section). Moisture (g water/100 g sample) was measured by drying a 3 g sample at 100 °C to constant weight [5], and the pH values were obtained using a MP 220 pH meter (Mettler-Toledo, Columbus, OH, USA).

The weight loss of “*Mortadella di Campotosto*”-like samples during drying was gravimetrically determined and calculated as shown in the following Equation (1):

Weight loss (%) = [(m0 − mt)/m0] × 100(1)

where m0 is the weight of sausage obtained after filling and mt is the weight of the sausages after a specific processing time (0, 6, 11, 18, 30, 45, and 120 days).

The measurements of moisture were performed by air oven drying [6], while for the NaCl content, the method of Volhard (ISO 1841-2: 1996) was used [7].

### 2.4. Chemical Determinations

Total nitrogen content (TN, % *w*/*w*) was determined by the Kjeldahl method, while proteins were obtained by multiplying TN × 6.25 [8]. Non-protein nitrogen content (NPN, % *w*/*w*) was measured by the precipitation of proteins with trichloroacetic acid, followed by determination of the nitrogen in the extract by the Kjeldahl method. Proteolysis Index (PI, %) was calculated as the ratio between NPN and TN (PI % = 100 × NPN × TN^−1^), as previously reported [9].

To evaluate the intensity of the primary proteolytic changes during the process, sarcoplasmic and myofibrillar proteins were extracted [10]. The protein concentrations were determined using Bradford reagent (Sigma-Aldrich, Milan, Italy) and bovine serum albumin (BSA, Sigma-Aldrich) as standard reference, according to Bradford (1976) [11]. Sodium dodecyl sulphate-polyacrylamide gel electrophoresis (SDS-PAGE) was used to analyze proteins [12] by Mini Protean III electrophoresis equipment (Bio-Rad, Segrate, Italy), as previously described [10]. The GS-800™ Calibrated Densitometer (Bio-Rad, Segrate, Italy) was used to quantify the relative abundance of each protein band.

Total amino acids were extracted and measured on 2 g of each sample, using the Cadmium-ninhydrin method [13]. For the extraction of the free amino acids, the method proposed by Berardo and colleagues [14] was followed and concentrations were determined by Reverse-phase high performance liquid chromatography (RP-HPLC), as previously reported [15], by using the Waters AccQ Tag method (Millipore Co-Operative, Milford, MA, USA). Amino acids were converted to stable fluorescent derivatives by reaction with AccQ·Fluor reagent (6-Aminoquinolyl-N-hydroxysuccinimidyl carbamate). RP-HPLC was performed using a Waters liquid chromatography system consisting of a Waters™ 626 pump, Waters™ 600 S controller, and Waters™ 717 S autosampler (Millipore Co-operative, Milford, MA, USA), by means of a Nova-Pak™ C18 column (4 μm, 3.9 × 4.6 mm), heated to 37 °C in a Shimadzu model CTO-10AC column oven. Elution was performed in a gradient of solvent A (Waters AccQ·Tag eluent A), solvent B (acetonitrile: Aldrich Chemical Co., Milan, Italy), and solvent C (20% methanol in Milli-Q water), prepared as follows: initial eluent 100% A; 99% A and 1% B at 0.5 min; 95% A and 5% B at 18 min; 91% A and 9% B at 19 min; 83% A and 17% B at 29.5 min; 60% B and 40% C at 33 min and held under these conditions for 20 min before returning to 100% A. The concentration of A was maintained at 100% up to 65 min, after which the gradient was changed to 60% B and 40% C for 35 min, before returning to the starting conditions. The single amino acids were identified by comparing their retention times with calibration standards. Peak areas were processed using Millennium 32 software v.4.0 (Waters, Milford, MA, USA).

### 2.5. Determination of Volatile Compounds

Volatile compounds were determined by solid phase micro-extraction coupled with gas chromatography mass spectrometry (SPME/GC-MS) [16] on 5 g of MCB or MCH at the selected sampling times. Volatile peaks identification was carried out by computer matching of mass spectral data with those of the compounds contained in the Agilent Hewlett-Packard NIST 98 and Wiley v. 6 mass spectral database. The volatile compounds content was expressed as relative percentage area.

### 2.6. Determination of Biogenic Amines

The following eight biogenic amines were detected, identified, and quantified: tryptamine (TRP), β-phenylethylamine (β-PHE), putrescine (PUT), cadaverine (CAD), histamine (HIS), tyramine (TYR), spermidine (SPD), and spermine (SPM).

The procedure of amines extraction and derivatization was carried out as described by Martuscelli et al. [17]: an aliquot of 2 g was homogenized (in Stomacher Lab blender 400, International PBI, Milan, Italy) with 10 mL of 5% trichloroacetic acid (TCA) and centrifuged (Hettich Zentrifugen, Tuttlingen, Germany) at a relative centrifugal force of 2325× *g* for 10 min; the supernatant was recovered and the extraction was performed with 5% TCA acid. The two acid extracts were mixed and made up to 50 mL with 5% TCA acid; the final acid extract was filtered through Whatman 54 paper (Carlo Erba, Milan, Italy). For derivatization of the samples, an aliquot of each acid extract (0.5 mL) was mixed with 150 μL of a saturated NaHCO_3_ solution and the pH was adjusted to 11.5 with about 150 μL NaOH 1.0 M. Dansyl chloride (Fluka, Milan, Italy) solution (2 mL of 10 mg/mL dansyl chloride/acetone) was added to the alkaline amine extract. Derivatized extracts were transferred to an incubator and kept for 60 min at 40 °C under agitation (195 stokes) (Dubnoff Bath-BSD/D, International PBI, Milano, Italy). The residual dansyl chloride was removed by adding 200 mL of 300 g/L ammonia solution (Carlo Erba). After 30 min at 20 ± 1 °C and protected from light, each sample was brought up to 5 mL with acetonitrile (Carlo Erba) and filtered through a 0.22 µm PTFE filter (Alltech, Sedriano, Italy).

Biogenic amines were determined, after extraction and derivatization, by high-performance liquid chromatography (HPLC) using an Agilent 1200 Series (Agilent Technologies, Milano, Italy). In a Spherisorb S30ODS Waters C18-2 column (3 μm, 150 mm × 4.6 mm ID), 10 μL of sample was injected with gradient elution, acetonitrile (solvent A), and water (solvent B) as follows: 0–1 min 35% B isocratic; 1–5 min, 35%–20% B linear; 5–6 min, 20%–10% linear B; 6–15 min, 10% B isocratic; 15–18 min, 35% linear B; 18–20 min, 35% B isocratic. Identification of the biogenic amines (BAs) was based on their retention times and BAs content was reported as mg/kg of product.

### 2.7. Texture Analysis

Textural properties were evaluated at room temperature (22 ± 2 °C) using an Instron Universal Testing Machine (mod. 5422, Instron LTD, Wycombe, UK) equipped with a 500 N load cell.

Slices (1 cm thick) were transversally cut from the central part of the sausage. Cubic samples (1 × 1 × 1 cm) were cut from the inner part of the slices placed between the lard and the casing. Samples were compressed by a plunger with a plane circular surface (35 mm diameter) using a crosshead speed of 0.5 mm/s. Two different tests were carried out for the textural characterization:

- Compression–relaxation test: samples were compressed by 30% of their initial height, then the run was stopped and the plunger was maintained at the maximum compression extension for 2 min, after which the load was removed. The maximum peak force in compression (N) was taken as a hardness index and the relaxation load was used to study the elastic behavior.
- Texture Profile Analysis (TPA) test: samples were submitted to a two-cycle compression test to 30% of their initial height in the first compression. After the first cycle, samples were left for 1 min to recover their deformation and then, the second cycle was started. Hardness (N), cohesiveness (J J^−1^), springiness (mm), and chewiness (N mm) were determined as previously described [18].

Each test was carried out on 10 samples of each batch (MCB and MCH). Since, in both tests, the experimental conditions in the first compression stage were the same, 20 samples were used for hardness calculation.

### 2.8. Experimental Design and Statistical Analysis

Two batches of sausages characterized by different casings were analyzed over time. In detail, three sausages per batch were randomly taken and analyzed at each ripening time (0, 6, 11, 18, 30, and 45 days). Time zero was considered as the time in which the batter was just wrapped in the casings, thus samples of batter (which was the same for the two batches) were taken just before being wrapped. All the data were subjected to two-way analysis of variance (ANOVA) to test the significance of individual (casing, ripening time) and interactive (casing × ripening time) effect. The model used for the two-way ANOVA is presented in Equation (2):

Y_ijk_ = μ + α_i_ + β_j_ + γ_ij_ + ε_ijk_ (2)

where μ is the intercept; α the casing factor; β the time factor; γ the interaction; ε the error; i and j are the level of the first and second factor; k the number of within group replicates. The significance of the effects was tested by Fisher’s F value and the associated *p* value.

Since the MCH batch was still not ripened after 45 days, an additional sampling was carried out only for this batch at 120 days. As the definitive experimental design was incomplete, data were further analyzed by the two-way nested ANOVA and the model used is presented in Equation (3):

Y_ijk_ = μ + α_i_ + β_j(i)_ + ε_ijk_ (3)

where β is the time factor nested with the casing factor. Post hoc mean comparison was carried out on the time nested with casing effect using the Tukey’s HSD test.

The mold load and textural properties of the fully ripened sausages (MCB at 45 days and MCH at 120 days) were eventually compared among them using the Student’s *t*-test for independent samples in order to test significant differences between the two groups of samples.

Statistical analyses were performed by using Statistica v. 6.1 (Statsoft Europe, Hamburg, Germany).

## 3. Results and Discussion

### 3.1. Effect of the Different Casings on Microbial Growth

Differently from other Mediterranean dry fermented sausages, in which the fermentation time is 1–2 days at 18–24 °C or 1 week at relatively low temperatures (10–12 °C), the fermentation of “*Mortadella di Campotosto*” sausages is carried out at very low temperatures, below 4 °C. These conditions, together with the absence of starters, cause a slow growth of lactic acid bacteria and therefore, an extension of the fermentation time.

Table 1 depicts the behavior of the different microbial groups during time. As evidenced, during the fermentation phase (up to 11 days) and the first week of ripening (day 18), no statistically significant differences were noticed in the growth dynamics of all microbial groups between MCB and MCH products. As expected, Enterobacteriaceae were not detected after 6 days of production, probably as a consequence of the progressive pH reduction. With the extension of the ripening time (from 30 days), statistically significant differences were observed in yeast and CNS counts that were lower in MCB. In detail, the yeast count increased in MCH samples at 30 days, probably because of a succession of different species. It has to be underlined that at 30 days, the a_w_ values of MCB and MCH samples were significantly different and the higher MCH a_w_ allowed a greater microbial growth with more abundant cells loads. After that, the number of cells progressively decreased during the ripening time.

In addition, in this study, we observed that the type of casing used can affect the colonization of molds, which reached values of 5.60 Log CFU/cm^2^ in MCB and < 2.0 Log CFU/cm^2^ in MCH at the end of the process.

### 3.2. Effect of the Different Casings on Physicochemical Parameters

In Table 1, the changes in pH values during fermentation and ripening of the samples in beef casing (MCB) and in hog casing (MCH) are reported. A significant (*p* < 0.05) pH decrease was detected in both cases up to day 11, which could thus be presumably considered as the end of fermentation. The pH decrease was concurrent with the increasing number of presumptive LAB that reached levels of 7.66 and 7.85 Log CFU/g for MCH and MCB, respectively. After that, pH slowly increased, due to the typical phenomena of ripening, starting with proteolysis in both batches, but with different rates throughout ripening. The end of ripening (45 days for MCB and 120 days for MCH, respectively) was first evaluated by professional manufacturers, who tested product hardness, as perceived by digital pressure, and flavor sniffing, and then, confirmed by textural analysis before final sampling. At the end of ripening, the pH reached levels of about 5.67 for MCB and of 5.90 for MCH, respectively.

**Figure 1:**
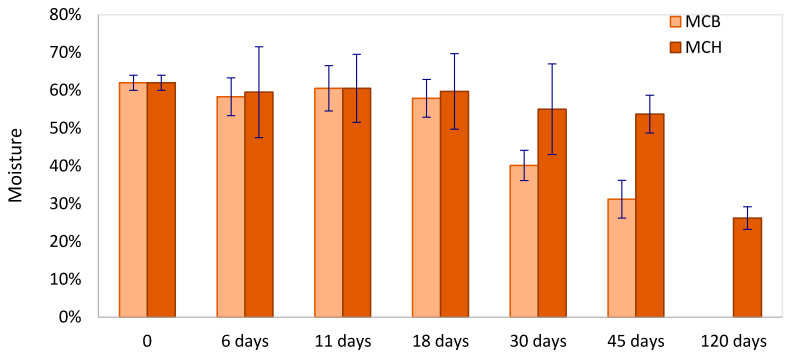
Evolution of the relative humidity of “*Mortadella di Campotosto*”-like sausages produced with beef (MCB) and hog (MCH) casings over time.

The type of casing exerted a significant effect on the drying rate, as highlighted by moisture (Figure 1) and a_w_ (Table 1) data; no water losses were observed during fermentation but, during ripening, the moisture dramatically differed between the two batches. After 45 days of ripening (the end of the ripening for MCB products), MCB batches reached moisture (31.2%) and a_w_ (0.841) values significantly lower than MCH samples, in which the values of moisture and a_w_ were 53.7% and 0.923, respectively. These differences can be attributed to the physical characteristics of the two types of casing, such as the degree of casing permeability, which influences the level of exchange between the filling and the external environment. In fact, hog bung casings had greater thickness (about 3-fold) than MCB, leading to a lower water vapor transmission rate and higher a_w_ [4]. The degree of casing permeability to water, gas, and light affects water loss, fat hydrolysis, fat oxidation, as well as pH and a_w_ [2,4].

As regards NaCl content, given as g 100/g total solids, slightly significant differences were observed at the end of the ripening time in both types of sausages, which showed values of 4.77 ± 0.01% and 4.97 ± 0.16% for MCB and MCH, respectively.

### 3.3. Effect on Proteolysis

**Figure 2:**
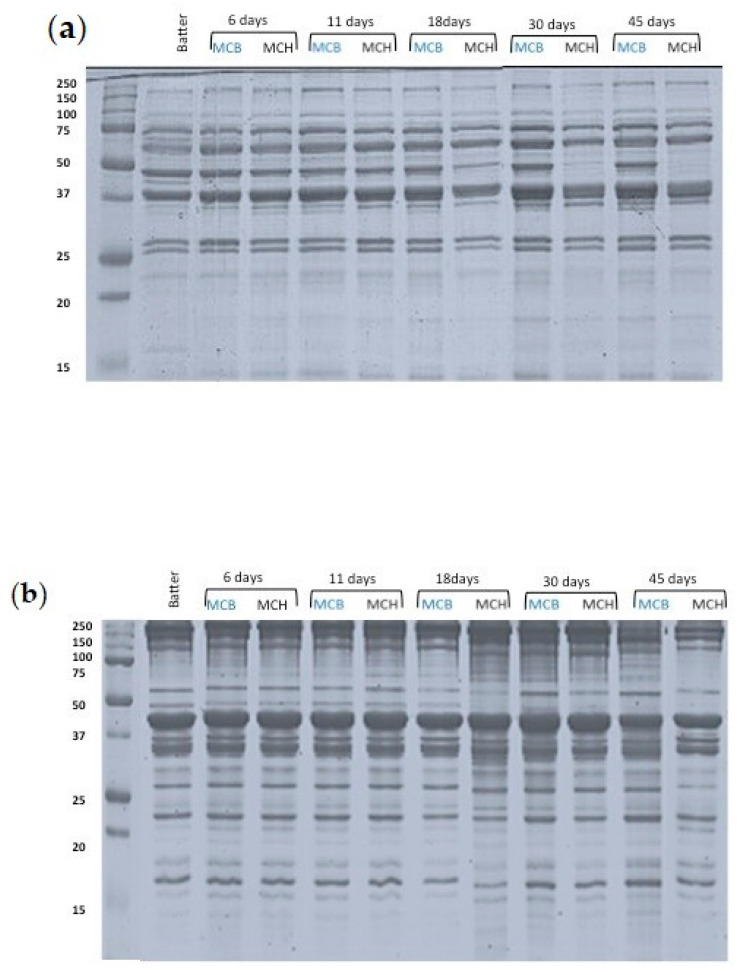
SDS-PAGE electrophoretic profiles of “*Mortadella di Campotosto*”-like sausages produced with beef (MCB) and hog (MCH) casings over time. Panel (**a**) sarcoplasmic fraction; panel (**b**) myofibrillar fraction. Batter: 0 days. Values on the ordinates refer to the marker and express the bands dimension in KDa.

Protein hydrolysis was evaluated by gel electrophoresis, as well as by measuring the content of total free amino acids, volatiles, and amines that greatly influence the texture, flavor, and safety of dry fermented sausages [19]. The hydrolysis of sarcoplasmic and myofibrillar proteins, determined via SDS-PAGE analysis, was influenced by the ripening time and the type of casing (Figure 2).

#### 3.3.1. Sarcoplasmic Fraction

The electrophoretic separation of sarcoplasmic proteins of MCB and MCH at different processing stages is illustrated in Figure 2a. Proteolysis took place during fermentation, as revealed by the slight changes from the first fermentation phase; the bands most susceptible to degradation were those of about 61 and 56 kDa, followed by a huge band of about 48 kDa, and the most intensive degradation was observed in MCH samples. In addition, two fragments were generated at 36 and 35 KDa, which were assumed to be glyceraldehyde-3-phosphate dehydrogenase [20], and 18 KDa in both types of samples.

In addition, during ripening, starting from day 18, a more intense hydrolysis was observed in MCH samples, as indicated by the intensity decrease in the band at 45 kDa (data at 120 days not shown), which is assumed to be creatine kinase [21]. This band completely disappeared after 45 days, while the intensity of the bands of about 74, 37, 36, 18, and 12 KDa increased overall in the MCB samples. The appearance and increase in polypeptides in the range of 14–100 kDa have been observed also by other authors [10,22].

As a_w_ strongly affects the activities of all endogenous proteinases [23], the differences between MCB and MCH could be ascribable to the higher a_w_ values of MCH samples (Table 1). Thus, it could be possible that the highest a_w_ values in MCH batches could have favored the activity of cathepsins B, which are able to break down sarcoplasmic proteins [24]. However, the contribution of bacterial enzymes to protein degradation needs to be taken into account, since LAB counts at day 30 reached higher values in MCH samples (8.24 Log CFU/g) than in MCB samples (6.48 Log CFU/g). In this context, in addition to LAB microbial enzymes, also *Staphylococcus carnosus* and *Staphylococcus simulans* proteases are capable to hydrolyze sarcoplasmic proteins [25].

#### 3.3.2. Myofibrillar Fraction

Myofibrillar proteins play the most critical role during meat processing, as they are responsible for the cohesive structure and firm texture of meat products [26]. As evidenced, this protein fraction was less susceptible to degradation and the hydrolysis dynamics in MCB and MCH showed very similar profiles. Recently, Berardo et al. [14] reported that actin (45 kDa) is highly broken down during fermentation; nevertheless, we did not evidence any change of this protein during fermentation in both MCB and MCH samples, in agreement with other studies [27,28]. On the contrary, the generation of polypeptides and large peptides with molecular weight from 50 to 100 kDa was more evident at day 11.

During ripening, when the endogenous enzymes are affected by the a_w_ decrease, having particular impact on cathepsins and alanyl- and pyroglutamyl-amino-peptidases [29], an important degradation of the band of about 48 kDa was detected (day 18); afterwards, it disappeared in both batches. In the meantime, a band of about 33 KDa, probably corresponding to β-tropomyosin, appeared in MCH samples at day 18, while in MCB samples, it was detected at 30 days of ripening [30].

Differences between the two products were clear from the 30th day of the process, in which the a_w_ values were 0.890 ± 0.008 and 0.940 ± 0.012 for MCB and MCH, respectively.

As regards Myosin Heavy Chain (220 kDa), an important degradation for at least 45% in MCH samples was detected at day 45, in contrast with MCB samples, in which it remained almost unchanged during ripening. The hydrolysis of actin (45 kDa) was less severe than that of myosin (25 kDa), and it was clear from day 30 in MCB, in which the reduction was about 15%. These results are in accordance with other authors [31], who reported a lower degradation of actin in fermented sausages with higher pH, suggesting that this might be due to the low optimum pH of cathepsin D-like muscle enzymes, playing a major role in actin hydrolysis.

Additionally, changes in tropomyosin (35 kDa) were more intense in MCB samples that presented also hydrolysis of the bands at 48 and 54 kDa, probably corresponding to desmin. Moreover, the myosin II short-chain (about 18 kDa) showed an intensity increase of approximatively 10% in MCH samples; muscle proteinases predominate in proteolysis evolution along the dry fermented sausage ripening, while those from bacteria mainly act during fermentation [32]. Nevertheless, the major proteolysis of the myofibrillar fractions in MCH (presenting a_w_ values of 0.890 at day 30) could be attributed to particular bacteria or yeast species present in the meat or in the casing, well adapted to the particular environment of this sausage and probably dominating during fermentation and ripening. Moreover, on the MCB casing surface, the molds, reaching loads of 5.60 Log CFU/g, could have promoted the greater proteolysis [33]. In fact, during ripening, when a_w_ decreases, the molds, and especially strains of *Aspergillus* and *Penicillium* genera, tend to dominate due to their capability to overcome xerophilic and halophilic conditions. *Penicillium chrysogenum* and *P. nalgiovense* contribute to proteolytic activities [34] and *Penicillium chrysogenum* Pg222 proteolytic enzymes show activity on the principal myofibrillar proteins, including actin, myosin, tropomyosin, and troponin [35].

### 3.4. Total Amino Acids Content

The generation of free amino acids (FAA) is the final outcome of proteolysis, and it contributes to the specific taste and also to the generation of volatile compounds, which provide the flavor in fermented sausages. The FAA content, expressed as mM of leucine, of both kinds of samples, was analyzed during the experimental period. As expected, the low temperatures applied in the production of the samples resulted in a limited generation of FAA, and their content was significantly different (*p* < 0.05) depending on the type of casing.

The quantification of total free amino acids (TFAA) is reported in Table 2. As evidenced, the initial batter contained about 361.25 ± 13.32 mg amino acids/100 g of dry-matter, and during the process, this concentration changed over time to a final concentration of 84.35 ± 19.18 and 235.59 ± 6.59 mg/100 g of dry-matter for MCB and MCH, respectively, at 45 and 120 days, with the major contribution of arginine (Arg) and alanine (Ala), followed by leucine (Leu) and valine (Val). The observed fluctuations in the content of each individual amino acid could be ascribed to the balance between FAA produced by protein breakdown and microbial activity. Among the bacteria, coagulase-negative staphylococci (CNS), *Lactobacillus sakei*, *Lactobacillus curvatus*, and some yeasts such as *Saccharomyces cerevisiae* have been reported to be directly involved in meat proteolysis and in free amino acids generation [15]. At the same time, many of these microorganisms use free amino acids as substrate for further metabolic reactions (deamination, dehydrogenation, and transamination), which are related to the development of aroma and flavor that characterize the final fermented sausage [36,37]. Moreover, arginine reduction in MCH samples could also be correlated to decarboxylation reactions, with the consequent production of putrescine, starting from day 45.

The major differences in FAA were observed during ripening, in which hydrophobic amino acids were accumulated. In addition, significant differences (*p* < 0.05) were observed for the greater amounts of Arg and Ala in MCB samples up to 30 days. This concentration appeared dramatically reduced at day 45, particularly for Arg in MCH samples and for Ala in MCB ones. In this respect, two hypotheses can be proposed: (1) the different environmental conditions present in MCB after 30 days could have selected microorganisms with a highly efficient arginine-converting machinery, with the aim of obtaining energy from arginine in the absence of glucose, as reported for some *Lactobacillus*, CNS and *Pseudomonas* species [38,39,40], and this might be reflected by the presence of *Pseudomonadaceae* at values of 5.11 ± 0.2 Log CFU/g in MCB samples at day 45; or (2) a possible oxidation of this amino acid could have happened by means of free radicals generated by lipolysis, leading to the formation of carbonyl groups [41]. In fact, amino acids such as lysine, threonine, arginine, and proline are easily attacked by these radicals.

In the case of MCH, the major FAA at the end of ripening was Ala with amounts of 123.20 mg/100 g of dry-matter.

### 3.5. Volatile Compounds Derived from Amino Acids

FAA are very important in fermented sausages, both for their contribution to the specific taste and for their involvement in degradation reactions that generate volatile compounds, which provide the flavor in this type of product. It is documented that the pH rise during fermentation is due to the microbial degradation of FAA by decarboxylation and deamination [42]. The transamination and decarboxylation of valine, isoleucine, and leucine, which are branched amino acids, produce the respective branched aldehydes, alcohols, and/or acids. Additionally, amino acids such as Phe, Thr, Try, Tyr, etc., are transformed into their respective aldehydes, such as phenylacetaldehyde from phenylalanine, and indole compounds from tryptophan, while the degradation of the sulfur amino acids cysteine and methionine produces sulfur volatile compounds [43]. In particular, we analyzed the accumulation dynamics of compounds derived from branched amino acids (branched aldehydes, alcohols, and carboxylic acids), such as 2-methylpropanal derived from valine (Val), 2-methylbutanal from isoleucine (Ile), 3-methylbutanal from leucine (Leu), and phenylacetaldehyde, benzaldehyde, phenylethyl alcohol, and ethyl benzoate ester that derived from phenylalanine (Phe) and the results are shown in Table 3.

In general, a greater relative abundance of branched chain aldehydes, alcohols, and acids from the catabolism of branched chain amino acids was detected in MCB samples. In particular, 3-Methyl-1-butanal was the most abundant compound in both types of samples, being more present during fermentation (up to 11 days) in MCH and during ripening in MCB samples. In this respect, other authors [44] suggested that 3-Methyl-butanoic acid and 3-Methyl-1-butanal are markers of the CNS activity in fermented meats.

On the other hand, the decrease in 3-Methyl butanol in MCB samples could be ascribed to its conversion into the corresponding 3-Methyl-butanoic acid (Table 3). MCB samples were characterized also by a major presence of 2-Methylpropanal and 2-Methylpropanoic acid, which increased over time and were not detected in MCH samples during the entire production period. On the contrary, phenyl ethyl alcohol concentration increased only in MCH batches.

### 3.6. Texture Analysis

The textural properties of “*Mortadella di Campotosto*”-like sausages were studied both by stress relaxation and TPA (Texture Profile) analysis. TPA parameters of the two types of samples at the end of ripening are shown in Table 4. At the end of ripening, corresponding to 45 days for MCB and 120 days for MCH, the two types of samples showed very similar textural profiles, except for small differences in springiness and chewiness.

No statistically significant differences were found in hardness, despite MCB showing lower a_w_ and moisture values and a slightly higher proteolysis index (12.92% vs. 12.13%) than MCH. In general, protein breakdown during fermented sausages ripening contributes to hardness decrease [45]. However, beside proteolysis, drying is a major factor affecting the binding and textural properties of fermented meat products. In most cases, the effect of dehydration, which increases hardness by promoting the elastic behavior, could counteract and even overcome the effect of proteolysis [28,46].

MCH samples showed a more elastic physical behavior and a consequently higher chewiness. Chewiness resulted positively affected by proteolysis in many studies, independently from positive or negative hardness changes [28,47,48]. Since in TPA analysis, chewiness is the product of hardness × cohesiveness × springiness, the higher chewiness of the MCH product observed in this study is due to its higher springiness, since no differences were found in hardness and cohesiveness. Springiness, which is a measure of elasticity [18], is depleted by moisture content, since water acts as a plasticizer and promotes viscous behavior. Despite differences in springiness, observed by measuring the recovery of the deformation after uniaxial compression, no significant differences in elastic behavior were observed when a stress–relaxation test was applied, as the force dissipated by viscous flow was identical in the two samples (Table 4).

In this section, the effect of proteolysis and dehydration were discussed since they are the main factors affecting sausage texture, but it should be considered that also lipolysis may contribute to the final texture.

### 3.7. Biogenic Amines (BA) Content

**Figure 3:**
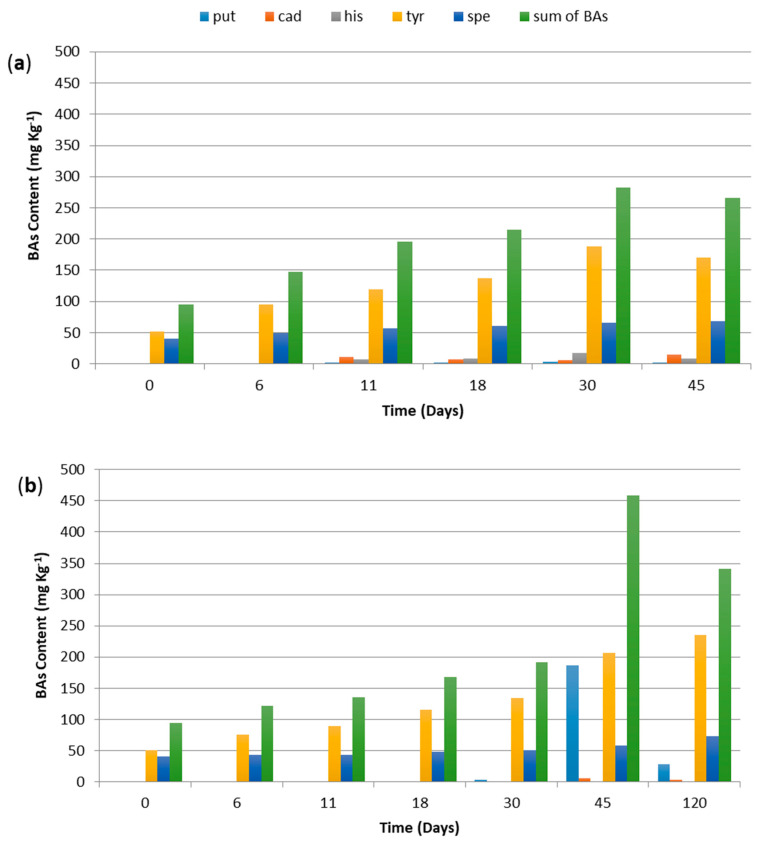
Putrescine (put), cadaverine (cad), histamine (his), tyramine (tyr), spermidine (spd), and sum of biogenic amines (as mg/Kg) content in “*Mortadella di Campotosto*”-like sausages produced with beef (panel (**a**), MCB) and hog (panel (**b**), MCH) casings, over time.

High quantities of proteins, associated with the proteolytic activity of endogenous enzymes and decarboxylase activity of wild microbiota, can support the accumulation of biogenic amines in fermented sausages [49,50], although the final balance depends on the equilibrium between BAs formation and degradation [51]. Figure 3 depicts the BAs content in “*Mortadella di Campotosto*”-like samples up to the end of ripening (45 days for MCB, panel a; 120 days for MCH, panel b).

In general, tryptamine, phenylethylamine, and spermine were not detected, while tyramine (TYR) and polyamines such as cadaverine (CAD), putrescine (PUT), and spermidine (SPD) were found during the entire production process, although with differences between the two products. Histamine was not detected in MCH, while it was found in MCB at low concentration (up to 17 mg/Kg, after the drying step). In addition, during fermentation (up to 11 days), TYR and SPD were the most abundant amines in MCB sausages and were detected in similar concentrations in MCB and MCH samples at up to 45 days of ripening. Tyramine production has been associated with the presence of LAB and enterococci that usually possess high amino acid decarboxylase activity [52]. This characteristic is strain-dependent and could be expressed during drying and ripening [53].

The sum of BAs content at the end of ripening resulted significantly different between the two types of products (265 ± 6 and 341 ± 23 mg/Kg, respectively, for MCB and MCH). These BAs levels are commonly found in other types of dry sausages produced by natural fermentation. Again, the differences in BAs content could be attributed to the higher water activity in MCH, as well as to the lower NaCl concentration due to scarce water loss, optimal for microbial development and BAs accumulation, in particular of tyramine and putrescine [54]. The significant reduction in total BAs at the end of ripening was associated with PUT decrease (more than 80%) in MCH samples, and with the decline of TYR, close to 40%, in MBH samples. Biogenic amines degradation was probably due to microorganisms possessing amino-oxidase enzymes, such as particular strains of the genera *Lactobacillus*, *Pediococcus*, *Micrococcus*, as well as *Staphylococcus carnosus* [55]. The activity of these enzymes is pH-dependent and particularly active at pH values close to neutrality [56].

In the absence of a legal limit for biogenic amines content in dry fermented sausages, European Food Safety Authority (EFSA) stated that up to 50 mg of histamine and 60 mg of tyramine can be considered safe for healthy individuals; however, these limits fall dramatically if an individual takes anti-MAO drugs or is particularly sensitive to these amines [57]. Suzzi and Gardini [53] identified a sum of 200 mg/Kg of vasoactive amines (tyramine, histamine, tryptamine, and 2-phenylethylamine), as an indicator of good manufacturing procedure for fermented sausages. Among the investigated samples, only in MCH this limit was reached 45 days after the start of the manufacturing process and was still exceeded (although reduced) at the end of ripening.

## 4. Conclusions

The effect of different casing on some characteristics of dry fermented sausages produced at very low temperatures was investigated. This study demonstrated that “*Mortadella di Campotosto*”-like sausages did not show an intense proteolysis during fermentation and ripening, probably because production is carried out at very low temperatures. Nevertheless, the type of casing had a strong effect on ripening time, proteolysis, and production of some volatile compounds. On the other hand, although the presence of biogenic amines is considered as unavoidable in fermented meat products, the results highlighted that in “*Mortadella di Campotosto*”-like sausages processed with the traditional beef casing, the risk associated with the presence of bioactive amines is low. Furthermore, in addition to the better texture performance, when beef casing is used for this type of sausage, the process is significantly shorter in comparison to hog casing, with important positive effects on production costs for ripening and storage. For all these considerations, and despite its lower availability on the market, beef casings, traditionally used for this kind of product, are determinant for the final characteristics of this type of product.

## Figures and Tables

**Table 1:** Physical-chemical and microbiological characteristics of “*Mortadella di Campotosto*”-like sausages produced with beef (MCB) and hog (MCH) casings over time.

					Microbial Groups (Log CFU/g)							
Batch	Time (Days)	pH	a_w_	MAB	Yeasts	LAB	LAC	ENTC	CNS	PSE		ENTB
**Complete design (CD)**												
MCB	0	5.96 ± 0.04 ^a^	0.957 ± 0.003 ^a^	7.24 ± 0.08 ^b^	7.14 ± 0.00 ^a^	6.37 ± 0.11 ^cd^	7.07 ± 0.06 ^bc^	6.52 ± 0.38 ^cd^	5.87 ± 0.36 ^abc^	5.56 ± 0.38 ^a^		3.09 ± 0.20 ^a^
MCH	0	5.86 ± 0.12 ^a^	0.959 ± 0.004 ^a^	7.12 ± 0.18 ^b^	7.02 ± 0.10 ^a^	6.60 ± 0.08 ^cd^	7.02 ± 0.10 ^bc^	6.92 ± 0.10 ^cd^	5.91 ± 0.48 ^abc^	5.71 ± 0.46 ^a^		3.42 ± 0.08 ^a^
MCB	6	5.50 ± 0.09 ^b^	0.956 ± 0.002 ^a^	7.55 ± 0.21 ^ab^	7.63 ± 0.13 ^a^	7.53 ± 0.18 ^abc^	7.80 ± 0.04 ^abc^	7.29 ± 0.13 ^cd^	6.98 ± 0.14 ^d^	4.72 ± 0.39 ^a^		0.95 ± 0.75 ^b^
MCH	6	5.47 ± 0.17 ^b^	0.956 ± 0.002 ^a^	7.17 ± 0.07 ^b^	7.63 ± 0.09 ^a^	7.14 ± 0.13 ^abcd^	8.13 ± 0.33 ^ab^	7.84 ± 0.04 ^b^	7.18 ± 0.13 ^a^	4.41 ± 0.61 ^a^		1.11 ± 0.31 ^b^
MCB	11	5.48 ± 0.07 ^b^	0.960 ± 0.006 ^a^	7.91 ± 0.09 ^ab^	7.80 ± 0.26 ^a^	7.85 ± 0.04 ^abc^	7.93 ± 0.09 ^ab^	7.79 ± 0.04 ^b^	6.29 ± 0.41 ^ab^	4.58 ± 0.49 ^a^		<1.00 ^b^
MCH	11	5.44 ± 0.08 ^b^	0.944 ± 0.009 ^a^	8.15 ± 0.33 ^ab^	7.26 ± 0.08 ^a^	7.66 ± 0.31 ^abcd^	8.28 ± 0.22 ^a^	8.09 ± 0.04 ^b^	4.47 ± 0.31 ^c^	4.45 ± 0.62 ^a^		<1.00 ^b^
MCB	18	5.62 ± 0.20 ^ab^	0.960 ± 0.006 ^a^	7.85 ± 0.21 ^ab^	4.00 ± 0.04 ^cd^	6.23 ± 0.27 ^d^	7.83 ± 0.20 ^abc^	7.64 ± 0.20 ^bc^	5.11 ± 0.15 ^bcd^	5.64 ± 0.01 ^ab^		<1.00 ^b^
MCH	18	5.55 ± 0.13 ^ab^	0.952 ± 0.006 ^a^	7.79 ± 0.12 ^ab^	4.00 ± 0.16 ^cd^	6.23 ± 0.27 ^d^	7.83 ± 0.26 ^abc^	7.64 ± 0.04 ^bc^	5.18 ± 0.15 ^bcd^	5.64 ± 0.27 ^ab^		<1.00 ^b^
MCB	30	5.65 ± 0.14 ^ab^	0.890 ± 0.008 ^b^	8.26 ± 0.15 ^ab^	4.80 ± 0.39 ^bc^	6.38 ± 0.06 ^d^	8.14 ± 0.13 ^ab^	8.03 ± 0.20 ^b^	6.22 ± 0.29 ^ab^	4.48 ± 0.95 ^ab^		<1.00 ^b^
MCH	30	5.56 ± 0.12 ^ab^	0.940 ± 0.012 ^a^	8.44 ± 0.20 ^a^	5.36 ± 0.17 ^b^	8.24 ± 0.20 ^a^	8.48 ± 0.39 ^a^	8.18 ± 0.08 ^b^	6.78 ± 0.29 ^ab^	3.95 ± 0.10 ^bc^		<1.00 ^b^
MCB	45	5.67 ± 0.11 ^ab^	0.841 ± 0.006 ^c^	8.08 ± 0.03 ^ab^	2.47 ± 0.20 ^e^	7.96 ± 0.22 ^ab^	8.54 ± 0.29 ^a^	6.31 ± 0.29 ^d^	2.00 ± 0.20 ^e^	5.11 ± 0.12 ^ab^		<1.00 ^b^
MCH	45	5.59 ± 0.09 ^ab^	0.923 ± 0.008 ^b^	7.97 ± 0.07 ^ab^	3.54 ± 0.22 ^e^	7.10 ± 0.14 ^abcd^	7.72 ± 0.39 ^abc^	7.76 ± 0.04 ^bc^	3.98 ± 0.23 ^d^	3.69 ± 0.10 ^bc^		<1.00 ^b^
F (C)		0.46	51.5 ***	0.20	93.5 ***	0.26	0.07	0.15	26.4 ***	2.90		0.06
F (t)		3.51 *	39.5 ***	9.10 ***	257 ***	1.7 ***	7.93 ***	12.3 ***	4.38 **	5.38 **		65.9 ***
F (C × t)		0.04	15.4 ***	0.66	25.6 ***	7.59 ***	1.68	5.16 **	34.5 ***	0.96		0.06
**Incomplete Design (CD + MCH 120 d)**												
MCH	120	5.90 ± 0.05 ^a^	0.812 ± 0.006 ^c^	7.55 ± 0.14 ^ab^		3.50 ± 0.14 ^d^	6.55 ± 0.36 ^b^	6.60 ± 0.34 ^c^	9.33 ± 0.11 ^a^	3.32 ± 0.06 ^de^	2.00 ± 0.20 ^c^	<1.00
F (C)		0.01	135 ***	0.47		151 ***	0.01	1.17	3.42	43.0 ***	12.3 **	0.26
F (t(C))		2.21 *	45.6 ***	4.60 ***		134 ***	7.77 ***	6.51 ***	13.3 ***	19.8 ***	6.41 ***	33.3 ***

Means with different letters in the same column are significantly different (*p* < 0.05). Log CFU/g—Log Colony Forming Unit/gram of product a_w_—water activity MAB—Mesophilic Aerobic Bacteria, LAB—Lactic acid bacteria; LAC—Lactococci; ENTC—Enterococci; PSE—Pseudomonas spp.; CNS—Coagulase negative Staphylococci; ENTB—Enterobacteriaceae. Fisher’s F value of casing (F(C)), time (F(t)), combined casing and time (F(C × t)), and time nested with casing (F(t(C))) factors calculated for each analytical determination. ANOVA significant differences were indicated by F values. * *p* < 0.05, ** *p* < 0.01. *** *p* < 0.005.

**Table 2:** Free amino acids content (mg/100 g meat) in “*Mortadella di Campotosto*”-like sausages produced with beef (MCB) and hog (MCH) casings over time.

Batch	Time (Days)	Arg	Asp	Glu	His	Ser	Ala	Gly	Ile	Leu	Met	Phe	Val	TOTAL
														FAA
Complete Design														
**MCB**	0	254 ± 2 ^a^	0.85 ± 0.04 ^ab^	10.1 ± 0.24 ^b^	n.d.	0.13 ± 0.08 ^bcde^	21.0 ± 2.25 ^d^	n.d.	2.36 ± 0.04 ^de^	7.80 ± 0.57 ^de^	59.4 ± 7.7 ^ab^	2.36 ± 0.20 ^f^	3.25 ± 0.20 ^bc^	361.25 ± 13.32 ^aa^
**MCH**	0	247 ± 2 ^a^	0.70 ± 0.16 ^ab^	11.8 ± 1.59 ^b^	n.d.	0.13 ± 0.08 ^bcde^	18.9 ± 0.53 ^d^	n.d.	2.20 ± 0.17 ^de^	6.70 ± 0.33 ^de^	58.4 ± 6.9 ^ab^	2.46 ± 0.13 ^f^	2.90 ± 0.08 ^bc^	351.19 ± 11.97 ^a^
**MCB**	6	192 ± 7 ^bcd^	0.69 ± 0.05 ^ab^	n.d.	n.d.	0.23 ± 0.03 ^bc^	4.50 ± 0.37 ^e^	n.d.	2.1 ± 0.17 ^de^	7.26 ± 0.30 ^de^	55.7 ± 0.5 ^b^	5.31 ± 3.10 ^bcd^	2.27 ± 0.11 ^c^	349.40 ± 10.10 ^a^
**MCH**	6	158 ± 23 ^d^	0.45 ± 0.07 ^bc^	n.d.	37.9 ± 0.87 ^b^	0.07 ± 0.01 ^cde^	6.81 ± 1.47 ^e^	37.9 ± 0.92 ^c^	1.31 ± 0.27 ^e^	5.46 ± 0.52 ^ef^	55.3 ± 3.2 ^b^	4.23 ± 0.39 ^de^	2.24 ± 0.18 ^c^	318.34 ± 15.66 ^ab^
**MCB**	11	236 ± 7 ^ab^	0.70 ± 0.05 ^ab^	1.73 ± 0.23 ^c^	n.d.	0.16 ± 0.01 ^bcde^	32.7 ± 2.5 ^c^	n.d.	2.46 ± 0.20 ^cd^	7.00 ± 0.15 ^de^	62.4 ± 1.8 ^ab^	3.56 ± 0.26 ^ef^	2.96 ± 0.37 ^bc^	270.34 ± 19.75 ^b^
**MCH**	11	220 ± 16 ^abc^	0.63 ± 0.30 ^ab^	2.80 ± 0.45 ^c^	2.50 ± 0.08 ^d^	0.09 ± 0.01 ^bcde^	17.8 ± 1.5 ^d^	3.95 ± 0.92 ^c^	1.53 ± 0.27 ^d^	6.26 ± 0.52 ^de^	55.6 ± 1.8 ^b^	3.73 ± 0.26 ^ef^	2.70 ± 0.38 ^bc^	310.07 ± 37.12 ^b^
**MCB**	18	177 ± 4 ^cd^	0.87 ± 0.08 ^ab^	n.d.	15.5 ± 1.16 ^c^	0.24 ± 0.02 ^bc^	136 ± 2 ^a^	15.5 ± 1.15 ^bc^	2.40 ± 0.15 ^cd^	8.44 ± 0.32 ^cde^	73.6 ± 0.2 ^a^	6.94 ± 0.17 ^ab^	2.57 ± 0.21 ^bc^	438.81 ± 6.61 ^c^
**MCH**	18	13.8 ± 0.9 ^e^	0.67 ± 0.07 ^ab^	n.d.	35.6 ± 1.74 ^b^	0.05 ± 0.01 ^de^	123 ± 4 ^b^	35.6 ± 2.57 ^a^	3.22 ± 0.10 ^bc^	14.3 ± 0.30 ^b^	7.20 ± 0.21 ^c^	3.42 ± 0.20 ^e^	3.11 ± 0.11 ^b^	237.78 ± 1.72 ^d^
**MCB**	30	186 ± 10 ^bcd^	0.95 ± 0.12 ^a^	n.d.	13.1 ± 1.02 ^c^	0.26 ± 0.01 ^b^	131 ± 1 ^ab^	13.1 ± 0.41 ^c^	3.35 ± 0.23 ^bc^	9.23 ± 0.37 ^cd^	21.8 ± 0.6 ^c^	8.42 ± 0.23 ^a^	2.56 ± 0.17 ^bc^	389.53 ± 20.13 ^a^
**MCH**	30	4.15 ± 0.90 ^f^	0.67 ± 0.06 ^ab^	n.d.	38.4 ± 1.24 ^ab^	1.07 ± 0.07 ^a^	128 ± 2 ^ab^	38.4 ± 2.02 ^a^	3.49 ± 0.25 ^ab^	13.4 ± 1.02 ^b^	7.07 ± 1.02 ^c^	4.31 ± 0.30 ^cde^	3.47 ± 0.18 ^b^	241.73 ± 9.05 ^b^
**MCB**	45	63.5 ± 15 ^e^	0.05 ± 0.11 ^cd^	n.d.	2.03 ± 0.21 ^d^	n.d.	1.61 ± 1.20 ^e^	2.03 ± 1.20 ^c^	3.61 ± 0.20 ^ab^	2.74 ± 0.18 ^f^	6.51 ± 0.88 ^c^	2.13 ± 0.20 ^f^	n.r.	84.35 ± 19.18 ^e^
**MCH**	45	8.43 ± 0.20 ^f^	0.97 ± 0.12 ^a^	15.9 ± 1.65 ^a^	45.9 ± 4.83 ^a^	0.14 ± 0.02 ^bcd^	135 ± 2 ^a^	32.9 ± 3.06 ^ab^	4.49 ± 0.19 ^a^	21.0 ± 1.52 ^a^	8.30 ± 0.41 ^c^	5.82 ± 0.12 ^bc^	5.16 ± 0.32 ^a^	287.32 ± 30.97 ^d^
F (C)		154 ***	0.12	48.8 ***	580 ***	34.5 ***	268 ***	109 ***	0.090	124 ***	65.3 ***	26.3 ***	76.9 ***	
F (t)		138 ***	3.58 *	96.2 ***	121 ***	143 ***	1808 ***	22.7 ***	45.5 ***	30.7 ***	119 ***	45.5 ***	4.87 **	
F (C × t)		27.8 ***	13.8 ***	41.9 ***	66.6 ***	98.6 ***	485 ***	10.7 ***	8.56 ***	63.0 ***	36.4 ***	43.3 ***	47.2 ***	
Incomplete design (CD + MCH 120d)														
**MCH**	120	1.61 ± 0.08 ^f^	n.r.	14.0 ± 0.70 ^ab^	33.0 ± 1.5 ^b^	1.10 ± 0.08 ^a^	123 ± 20 ^ab^	33.0 ± 2.5^a^	3.88 ± 0.20 ^ab^	11.4 ± 0.7 ^bc^	6.70 ± 0.67 ^c^	3.31 ± 0.26 ^ef^	4.75 ± 0.29^a^	235.59 ± 6.6 ^d^
F (C)		263 ***	0.06	112 ***	690 ***	125 ***	596 ***	133 ***	3.60	131 ***	120 ***	34.3 ***	111 ***	
F (t(C))		90.7 ***	0.29 ***	75.8 ***	87.6 ***	113 ***	1130 ***	16.0 ***	27.1 ***	42.1 ***	81.7 ***	41.3 ***	25.6 ***	

Arg—arginine; Asp—aspartic acid; Glu—glutamine; His—histidine; Ser—serine; Ala—alanine; Gly—glycine; Ile—isoleucine; Leu—leucine; Met—methionine Phe—phenylalanine; Val—valine; FAA—free amino acids. Means with different letters in the same column are significantly different (*p* < 0.05). Fisher’s F value of casing (F(C)), time (F(t)), combined casing and time (F(C × t)), and time nested with casing (F(t(C))) factors calculated for each analytical determination. ANOVA significant differences were indicated by F values. ** *p* <0.01. *** *p* < 0.005. n.d.—not detectable.

**Table 3:** Relative abundance of branched chain compounds derived from branched chain amino acids in “*Mortadella di Campotosto*”-like sausages produced with beef (MCB) and hog (MCH) casings over time. Results are expressed as relative abundance × 10^6^.

	Time (Days)	2-MPA (%)	3-MBA (%)	3-MB (%)	PEA (%)	3-M1-B (%)	2-MP (%)
**Complete Design (CD)**							
MCB	0	0.26 ± 0.01 ^de^	1.18 ± 0.02 ^de^	1.29 ± 0.07 ^c^	1.63 ± 0.50 ^bc^	7.93 ± 1.23 ^bc^	0.49 ± 0.01 ^d^
MCH	0	0.24 ± 0.01 ^de^	1.18 ± 0.02 ^de^	1.09 ± 0.09 ^c^	1.49 ± 0.62 ^bc^	7.93 ± 1.25 ^bc^	0.52 ± 0.01 ^d^
MCB	6	0.43 ± 0.03 ^cd^	2.90 ± 0.16 ^abcd^	7.37 ± 1.14 ^a^	0.15 ± 0.01 ^c^	2.64 ± 0.37 ^e^	0.2 ± 0.03 ^d^
MCH	6	n.d.	1.20 ± 0.20 ^cde^	2.81 ± 0.48 ^bc^	1.29 ± 0.26 ^bc^	7.89 ± 0.77 ^bcd^	n.d.
MCB	11	0.71 ± 0.07 ^bc^	3.6 ± 0.49 ^ab^	5.50 ± 1.17 ^ab^	0.19 ± 0.04 ^c^	5.42 ± 0.49 ^cde^	0.7 ± 0.05 ^d^
MCH	11	n.d.	1.50 ± 0.34 ^bcde^	2.40 ± 0.62 ^bc^	1.30 ± 0.29 ^bc^	9.2 ± 0.68 ^abc^	n.d.
MCB	18	0.88 ± 0.06 ^ab^	4.75 ± 0.95 ^a^	5.23 ± 1.76 ^ab^	0.23 ± 0.04 ^c^	12.7 ± 0.85 ^a^	1.7 ± 0.34 ^c^
MCH	18	0.26 ± 0.02 ^de^	0.60 ± 0.10 ^e^	0.2 ± 0.06 ^c^	1.28 ± 0.23 ^bc^	5.43 ± 1.09 ^cde^	n.d.
MCB	30	n.d.	3.88 ± 0.10 ^a^	n.d.	0.26 ± 0.03 ^c^	10.94 ± 0.84 ^ab^	2.8 ± 0.17 ^b^
MCH	30	1.23 ± 0.13 ^a^	0.65 ± 0.35 ^e^	n.d.	1.32 ± 0.43 ^bc^	7.63 ± 0.88 ^bcd^	n.d.
MCB	45	n.d.	3.83 ± 0.12 ^a^	n.d.	0.29 ± 0.05 ^c^	7.32 ± 1.02 ^bcd^	4.2 ± 0.53 ^a^
MCH	45	1.01 ± 0.16 ^ab^	1.07 ± 0.80 ^de^	n.d.	4.33 ± 0.83 ^a^	6.6 ± 0.31 ^bcde^	n.d.d
F (C)		4.28 *	88.7 ***	25.5 ***	48.7 ***	0.45	206 ***
F (t)		14.9 ***	9.08 ***	17.0 ***	7.11 ***	6.06 ***	32.2 ***
F (C × t)		97.6 ***	5.54 **	5.48 **	8.21 ***	14.5 ***	35.9 ***
**Incomplete Design (CD + MCH 120 d)**							
MCH	120	n.d.	3.44 ± 0.11 ^abc^	n.d.	2.15 ± 0.20 ^b^	3.7 ± 0.34 ^de^	n.d.
F (C)		0.05	67.7 ***	34.3 ***	58.3 ***	3.68	242 ***
F (t(C))		60.2 ***	9.45 ***	11.2 ***	7.39 ***	11.7 ***	33.5 ***

2-MPA-2-Methylpropanoic acid; 3-MBA-3-Methylbutanoic acid; 3-MB-3-Methylbutanol; PEA-phenyl ethyl alcohol; 3-M1-B-3-Methyl-1-butanal; 2-MP-2-Methylpropanal. Means with different letters in the same column are significantly different (*p* < 0.05). Fisher’s F value of casing (F(C)), time (F(t)), combined casing and time (F(C × t)), and time nested with casing (F(t(C))) factors calculated for each analytical determination. ANOVA significant differences were indicated by F values. * *p* < 0.05, ** *p* < 0.01. *** *p* < 0.005; n.d.—not detectable.

**Table 4:** Results of texture profile analysis and compression–relaxation test of ripened “*Mortadella di Campotosto*”-like sausages produced with beef (MCB) and hog (MCH) casings.

Product	Hardness (N)	Springiness (mm)	Cohesiveness (J × J^−1^)	Chewiness (N mm)	Relaxation Load (%)
MCB (45 days)	16.0 ± 3.4 ^a^	1.91 ± 0.19 ^b^	0.50 ± 0.04 ^a^	15.2 ± 2.9 ^b^	63.68 ± 0.63 ^a^
MCH (120 days)	16.5 ± 1.7 ^a^	2.06 ± 0.12 ^a^	0.52 ± 0.04 ^a^	17.6 ± 1.4 ^a^	62.70 ± 0.82 ^a^

Data in the same column with different letters are significantly different at a *p* < 0.05 level.
